# Differential binding affinity of tau repeat region R2 with neuronal-specific β-tubulin isotypes

**DOI:** 10.1038/s41598-019-47249-7

**Published:** 2019-07-25

**Authors:** Vishwambhar Vishnu Bhandare, Bajarang Vasant Kumbhar, Ambarish Kunwar

**Affiliations:** Department of Biosciences and Bioengineering, Indian Institute of Technology Bombay, Powai, Mumbai, 400076 Maharashtra India

**Keywords:** Protein structure predictions, Intrinsically disordered proteins, Computational biophysics

## Abstract

Tau is a microtubule-associated protein whose C-terminal domain consisting of four repeat regions R1, R2, R3 and R4 binds to microtubules to stabilize them. In several neurodegenerative diseases, tau detaches from microtubules to form insoluble aggregates leading to tauopathy. Microtubules are made up of αβ tubulin subunits. Seven α-tubulin and nine β-tubulin isotypes have been reported to be present in humans till date. These tubulin isotypes show residue composition variations mainly at C-terminal region and bind to motor proteins and anti-mitotic drugs differently. These tubulin isotypes show tissue specific expression as their relative proportion varies significantly in different type of cells. It is also known that tau binds differently to different cell lines and can either promote or demote microtubule polymerization. However, the relative binding affinity of tau to the different β-tubulin isotypes present in different cell lines is completely unknown. Here, we study relative binding affinity of Tau repeat region R2 to neuronal specific tubulin isotypes βI, βIIb, and βIII using molecular modelling approach. The order of binding energy of tau with tubulin is βIII > βIIb > βI. Our strategy can be potentially adapted to understand differential binding affinity of tau towards β-tubulin isotypes present in other cell lines.

## Introduction

**Figure 1:**
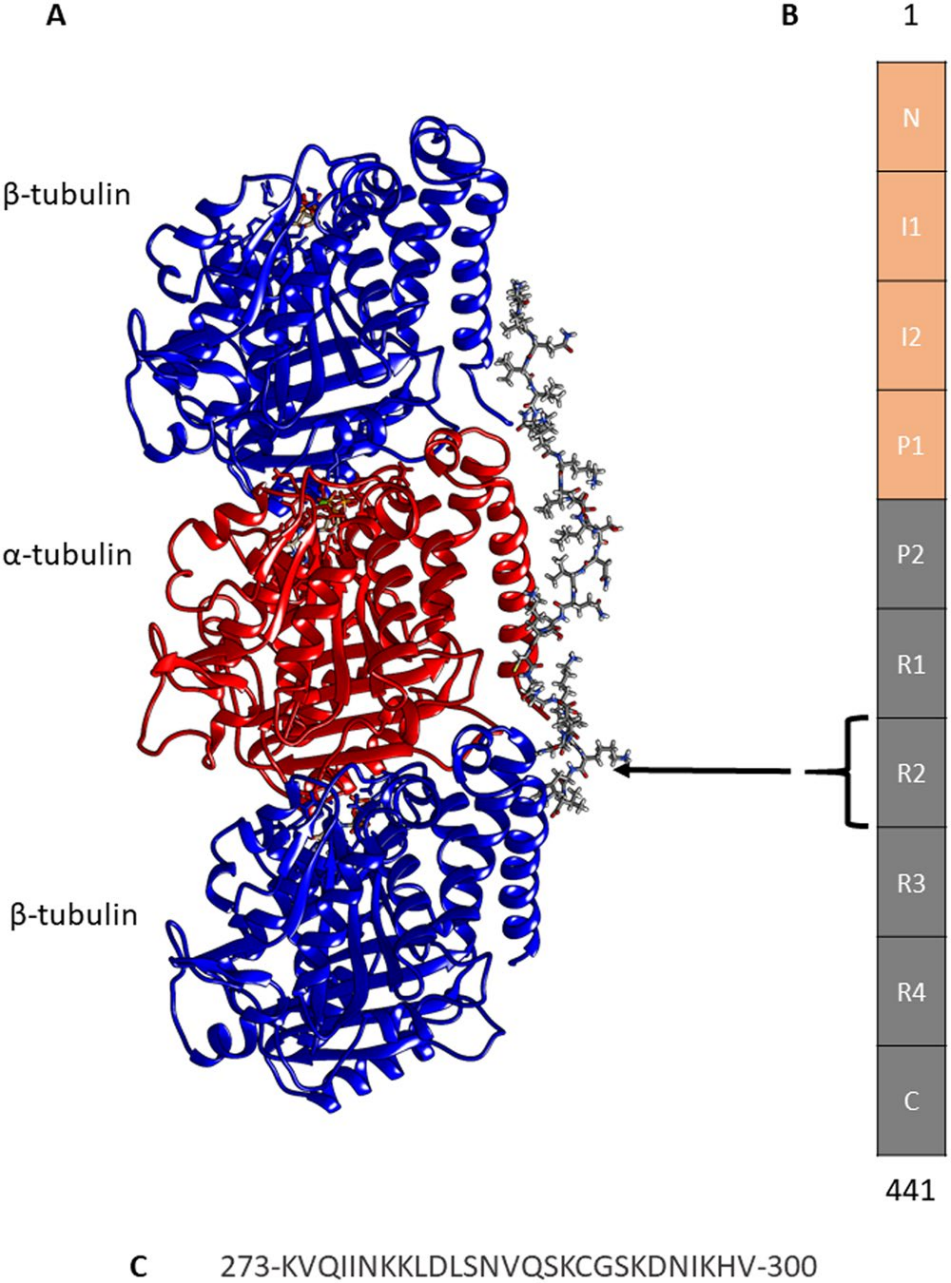
CryoEM Structure of tubulin subunits bound to TauR2. (**A**) tubulin subunits bound to TauR2 in CryoEM structure 6CVN.pdb. TauR2 domain binds at the outer surface of the MT. (**B**) Domain organization in tau, (**C**) sequence of TauR2.

Tau is a microtubule-associated intrinsically disordered protein encoded by gene ‘*mapt’* located on chromosome 17^1^, which is abundantly expressed in the brain and neuronal tissues^2,3^. A total of 6 tau isoforms are found in the human central nervous system whose length varies from 352 to 441 residues^4^. Tau consists of the N-terminal projectile domain (residue 1–244) which is composed of the acidic and proline-rich region, and the C-terminal domain comprising residues 245–441 which consists of 4 repeat regions i.e. R1, R2, R3 and R4 (Fig. 1). The isoforms of tau mainly differ by the presence of either R3 or R4 repeats at the C-terminal domain^5^. The longest isoform of tau is observed in humans which comprises 4 repeats i.e. R1, R2, R3 and R4. Whereas, the shortest isoform has 3 repeats (R1, R2 and R3) which is reported in the fetus brain^4,6^. Figure 1A shows the structure of tau repeat R2 bound to the β/α/β tubulin subunits in the recently released CryoEM model^7^. Hereafter, we shall refer to tau repeat R2 as ‘TauR2’. These tau repeats prefer to bind at the exterior surface of microtubule (MT) and help in its stabilization (Fig. 1A) and also regulate MT polymerization^5^. Figure 1B shows domain organization in the tau structure and Fig. 1C shows the sequence of TauR2. It is well known that tau mainly helps in the assembly and stabilization of axonal MTs, which contributes to the proper functioning of neuronal cells^8^. However, recently it has been reported that the tau is not only a stabilizer of axonal MTs but it is also enriched on the labile domain of the MT to promote its assembly^9^. In various neurodegenerative diseases, tau detaches from the MTs and forms abnormal, fibrillar structures of insoluble aggregates due to post-translational modifications^10,11^.

Structure of full-length tau has not yet been solved by using X-ray crystallography due to its intrinsically disordered nature. Also, efforts to obtain a solution structure of complete tau using NMR techniques have failed^12^. Thus, the interaction of tau repeats with MT has been studied using various biochemical and biophysical techniques^13–16^. Further, Cryo-EM study shows discontinuous density of tau repeat along with each protofilament upon MT binding^7^. Hence, the interaction of a synthetically developed R1 and R2 repeats of tau with MT were examined. It is observed that R1 and R2 repeat of tau adapt the extended structures along the crest of protofilament, stabilizing the interface between tubulin dimers^7^.

MTs are made up of αβ-tubulin heterodimers^17^. In eukaryotes, seven α-tubulin and nine β-tubulin isotypes have been reported which show tissue-specific expressions. For example, βI isotype is expressed in all cells, βII and βIII isotype are predominantly expressed in brain and neuronal cells, and βVI is expressed in erythroid cells and platelets^18^. βI plays important role in cell viability, βII is important for neurite growth and βIII protects neurons against free radicals and reactive oxygen species^19^. It has been observed that all the β-tubulin isotypes share a significant homology except at their C-terminal tail region^20–23^ which is highly flexible and disordered structure. The C-terminal tail regions in these isotypes protrude in the outward direction of the MTs where they interact with MAPs and regulate MT dynamics^24,25^.

It is well known that the composition of β-tubulin isotypes affect MT dynamic instability^22,26^, their interaction with motor proteins^27^, binding of anti-mitotic drugs^20,21,28^ and different MAPs including tau^29,30^. These tubulin isotypes show tissue specific expression as their relative proportion varies significantly in different type of cells^19,31,32^. It is also known that tau binds differently to different cell lines and can either promote or demote microtubule polymerization^33^. However, the relative binding affinity of tau to the different β-tubulin isotypes present in different cell lines is completely unknown. Here, we study relative binding affinity of Tau repeat region R2 to neuronal specific β-tubulin isotypes namely βI, βIIb, and βIII using molecular modeling approach.

## Results and Discussion

To gain insight into the detailed binding mode, relative binding affinity and inter-molecular interactions between neuronal specific tubulin isotypes and TauR2, we employ sequence analysis, homology modeling, MD simulations, and binding energy calculation.

### Sequence analysis and molecular modelling of tubulin isotypes

**Figure 2:**
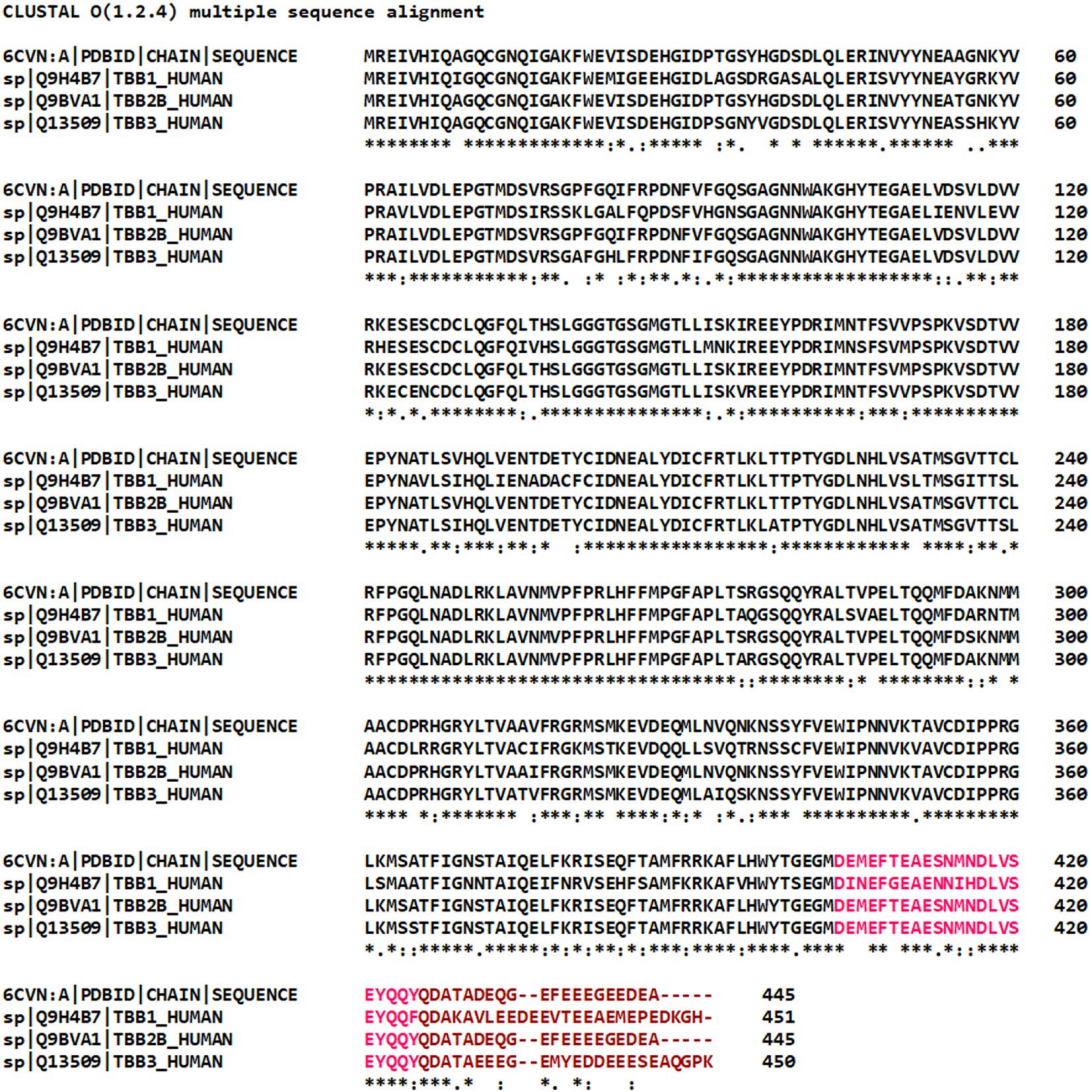
Multiple sequence analysis of different β-tubulin isotypes. The βI, βIIb, βIII tubulin isotypes and template 6CVN show maximum residue variations mainly at C-terminal tail region. The TauR2 binding regions H12 helix and C-terminal tail region of β-tubulin subunits are shown in hot pink and brown respectively.

The human β-tubulin isotypes show residue variations mostly at the carboxy-terminal tail region in multiple sequence alignment as shown in Fig. 2. The C-terminal tail regions of βI and βIII tubulin isotypes are longer when compared with the βIIb isotype. The template β-tubulin sequence (6CVN; chain A) and human βIIb isotypes show 98.65% sequence identity. These sequence variations in the tubulin isotypes are known to regulate MT protofilament number and their stability^34^.

These sequences of β-tubulin isotypes were used to build three-dimensional homology models using 6CVN as the template structure. The tubulin isotypes βI, βIIb, βIII were modeled using Modeller 9v20^35^. Best homology models for different tubulin isotypes were selected using DOPE score for chain A and chain C of β tubulins. The DOPE scores for the selected models of βI, βIIb and βIII isotypes are given in Supplementary Table S1. The assessment of stereo-chemical properties of homology models was done using Ramachandran plot^36,37^.

In Ramachandran plots for all the modeled β-tubulin isotypes, more than 98% of the residues occupy a favoured region. The occupancy of residues in different regions of the Ramachandran plot is listed in Supplementary Table S2. Quality of these selected homology models was further validated using GMQE score^38^, Verify3D^39^ and ERRAT score^40^ (Supplementary Table S3). The GMQE score provides an estimate of the accuracy of the tertiary structure of the modeled structures. The GMQE score for all the modeled β-isotypes was 0.98 which represents the accuracy of modeled structures of β-isotypes as its value was well above 0.7 considered as reliable. This was further confirmed by calculating Verify3D and ERRAT scores for the modeled structures. (Supplementary Table S3). These modeled structures of β-tubulin isotypes were further used to build the different tubulin and TauR2 complexes such as βI/α/βI-TauR2, βIIb/α/βIIb-TauR2 and βIII/α/βIII-TauR2 using 6CVN.pdb as a template. These modeled structures were further used as starting structures for MD simulations.

### Structural stability of the tubulin-TauR2 complexes

The MD simulations were performed on tubulin-TauR2 complexes such as 6CVN-TauR2, 6CVN*-TauR2, βI/α/βI-TauR2, βIIb/α/βIIb-TauR2, βIII/α/βIII- TauR2 using Gromacs 2018.1^41^. The stability of the simulated systems was accessed by plotting the potential energy during the course of simulation which revealed that the simulated tau-tubulin complexes were stable with well minimized energies (Supplementary Fig. S1).

**Figure 3:**
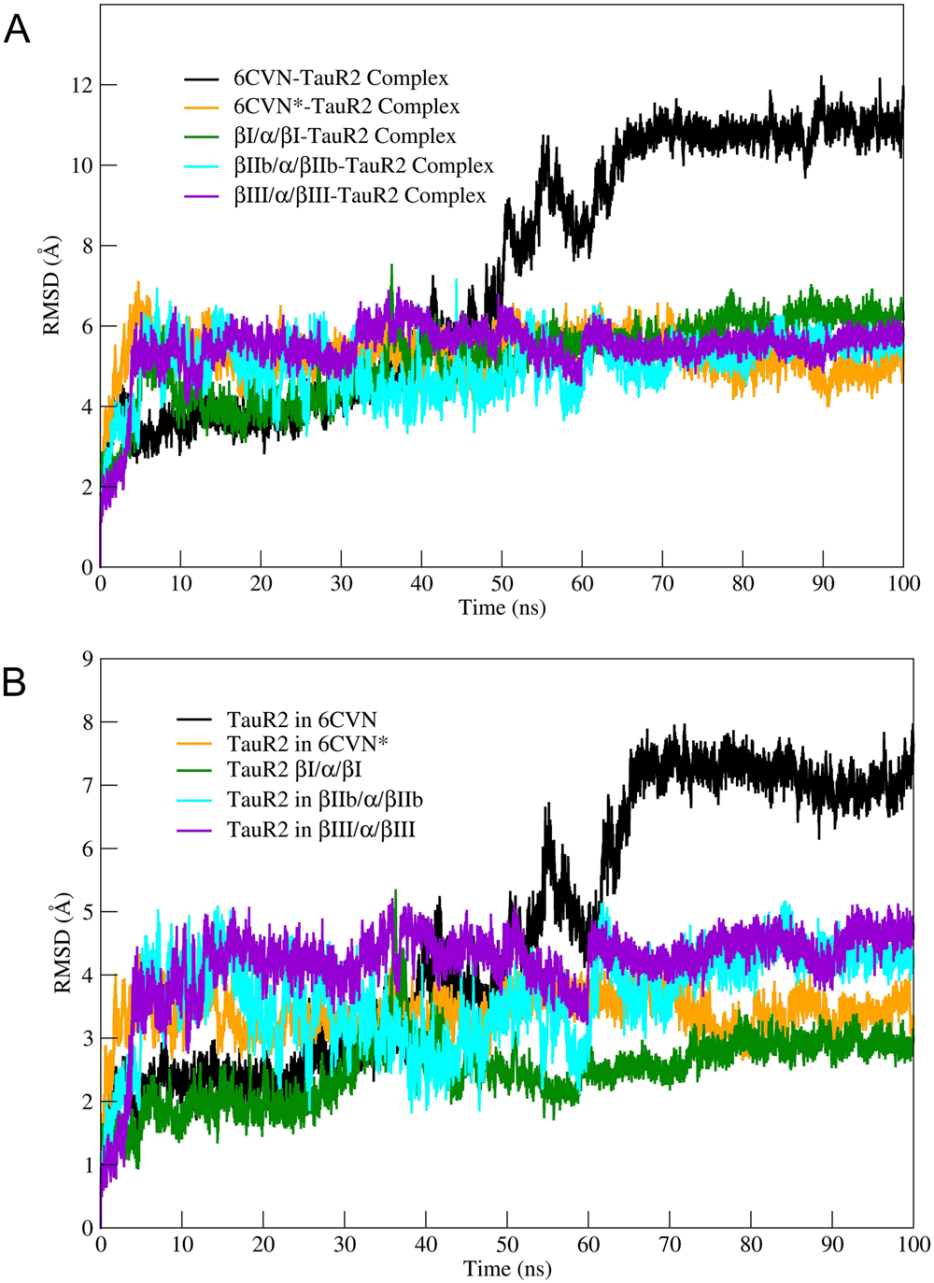
Stability of the tubulin-TauR2 complex and TauR2. (**A**) The Root mean square deviation values (RMSD) for tubulin-tauR2 complexes. RMSD values for 6CVN, 6CVN*, βI/α/βI, βIIb/α/βIIb and βIII/α/βIII have been plotted in black, orange, green, cyan and violet respectively. (**B**) The Root mean square deviation values for TauR2 shown using same color Scheme as in (**A**).

The stability of tau-tubulin complex was further accessed by calculating root mean square deviations (RMSD), root mean square fluctuations (RMSF), and radius of gyration (R*g*). The RMSD values for tubulin-TauR2 complexes, tau and backbone atoms of tubulin trimer (without considering flexible C-tail) plotted over the trajectory reveal the stability of all the complexes over the entire simulation period of 100 ns. The RMSD plot for tubulin-TauR2 complexes and TauR2 is shown in Fig. 3A, B respectively. The RMSD for the βIII/α/βIII-TauR2 complex is comparatively more stable than other tubulin-TauR2 complexes. Similarly, TauR2 bound to βIII/α/βIII shows stable dynamics during simulation. The complex 6CVN-TauR2 is stabilized at higher RMSD mainly due to absence of C-tail region which highlights the importance of C-terminal tail in the stabilizing tubulin-TauR2 complex.

All above simulated systems are well equilibrated with the backbone RMSD value of ~3.5 Å (Supplementary Fig. S2). The stable dynamics of all the simulated systems namely 6CVN-TauR2, 6CVN*-TauR2, βI/α/βI-TauR2, βIIb/α/βIIb-TauR2 and βIII/α/βIII-TauR2 are shown in Supplementary Movies S1–S5 respectively. To check the specificity of TauR2 towards tubulin subunits we replaced TauR2 with negative control polyA peptide of same length. Interestingly, our negative control system 6CVN*-polyA complex shows the loose binding during the simulation due to weaker interactions of polyA peptide with tubulin subunits (Supplementary Movie S6), which establishes that TauR2 has specificity towards tubulin subunits.

### Residue fluctuations of tubulin subunits and TauR2

The flexibility of tubulin trimers systems and TauR2 has been studied by plotting Cα fluctuations during the course of simulation. The RMSF for tubulin subunits and TauR2 is shown in Supplementary Fig. S3. TauR2 interacting residues from the H12 helix of β-tubulin and the C-terminal tail region (residue 400–451) show significant decrease in the fluctuations, as their free dynamics is arrested upon tau binding (Supplementary Fig. S3A,B). RMSF values for the tubulin β-subunits in the systems 6CVN*, βIIb/α/βIIb and βIII/α/βIII are lesser than those of 6CVN and βI/α/βI tubulin subunits (Supplementary Fig. S3B). The data also highlights the binding of TauR2 at the interdimer interface where it shows much lesser fluctuations while part of C-tail region which has no contact with TauR2 is highly flexible (Supplementary Fig. S3B). The H12 helix and C-terminal tail region contribute towards stronger binding by non-covalent interactions whose detailed analysis is given in the section ‘*Intermolecular interactions between tubulin and tau’*.

Further, we also analyzed the RMSF plot for TauR2 (Supplementary Fig. S3C) to analyze Cα-fluctuations of TauR2 for understanding its conformational behavior during MD simulations. It is observed that the TauR2 shows highest fluctuations at the N- and C-terminal region in the 6CVN, where the C-terminal tail region is absent (Supplementary Fig. S3C). Interestingly, residual fluctuations in TauR2 bound to βIII/α/βIII-tau complex are less when compared to other tubulin-TauR2 complexes such as 6CVN*-TauR2, βI/α/βI-TauR2 and βIIb/α/βIIb-TauR2 (Supplementary Fig. S3C). This also confirms that the C-terminal tail region of tubulin subunits plays an important role in the binding of TauR2.

Overall, RMSF suggests the importance of H12-helix and C-terminal tail region in the binding of TauR2 and greater affinity of TauR2 towards βIII tubulin isotypes. We further analyzed the compactness of all the tubulin-TauR2 systems using the radius of gyration (*R*_*g*_) as discussed in the next section.

### Compactness of tubulin-TauR2 complexes

**Figure 4:**
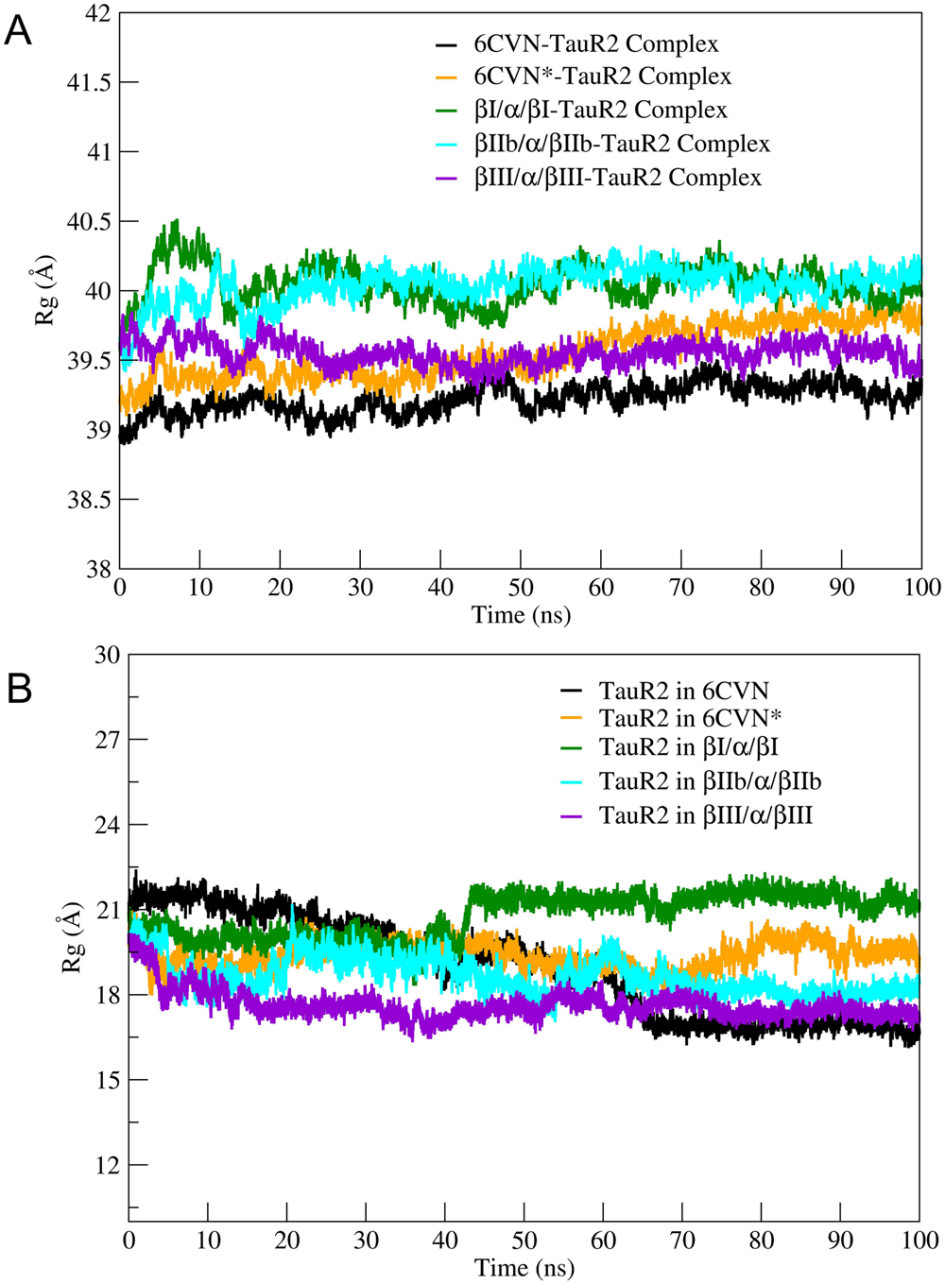
Radius of Gyration (R_*g*_) of different tubulin-TauR2 complexes and TauR2. (**A**) R_*g*_ of 6CVN-TauR2 (black), 6CVN*-TauR2 (orange), βI/α/βI-TauR2 (green), βIIb/α/βIIb-TauR2 (cyan), βIII/α/βIII-TauR2 (violet) (**B**) R_*g*_ for TauR2 in different tubulin-TauR2 complexes. Color scheme same as Fig. 3.

The radius of gyration (*R*_*g*_) indicates the level of compactness of the protein system which is helpful in getting an insight into the stability of the protein-protein complex. The tubulin-TauR2 complex shows the R_*g*_ values ranging from 38.8–40.5 Å (Fig. 4A). The complex βIII/α/βIII-TauR2 expresses stable R_*g*_ value throughout the simulation whereas 6CVN-TauR2, 6CVN*-TauR2, βI/α/βI-TauR2, βIIb/α/βIIb-TauR2 complexes show variations in their R_*g*_ values. Complex 6CVN-TauR2 shows less R_*g*_ values compared to other tubulin-TauR2 due to the absence of C-terminal tail region (Fig. 4A).

The *R*_*g*_ values of only TauR2 in different tubulin subunits are plotted in Fig. 4B for different tubulin-TauR2 complexes. The R_*g*_ values for TauR2 in case of 6CVN*, βIIb/α/βIIb, and βIII/α/βIII complexes shows fluctuations between 17.5 to 20 Å except for βI/α/βI complex (Fig. 4B). The βIII tubulin subunits shows *R*_*g*_ value of ~18 Å and βI tubulin subunits have largest *R*_*g*_ value of 22.5 as shown in Fig. 4B.

However, 6CVN bound TauR2 shows continuous decrease in *R*_*g*_ values from 21.5 Å to 16.5 Å. This suggests the importance of C-terminal tail region in the stable binding of tau (Fig. 4B).

It is important to note that βIII tubulin subunits (Supplementary Fig. S4) have *R*_*g*_ values similar to that of βIII/α/βIII-TauR2 complex (Fig. 4A). This implies that the tubulin subunits composed of βIII tubulin isotype are structurally stable after binding of TauR2.

Thus, our analysis of R_*g*_ for tubulin-TauR2 complexes, tubulin subunits and TauR2 highlights the stability of the βIII/α/βIII-tau complex over other tubulin-TauR2 complexes and importance of the C-terminal tail region in the binding of TauR2.

To understand the exposure of the interface residues of tubulin subunits bound to the TauR2, we studied contact surface area (CSA) and solvent accessible surface area (SASA) using ‘gmx sasa’ tool of gromacs software^42^.

### Solvent accessible surface area for tubulin-TauR2 complexes

**Figure 5:**
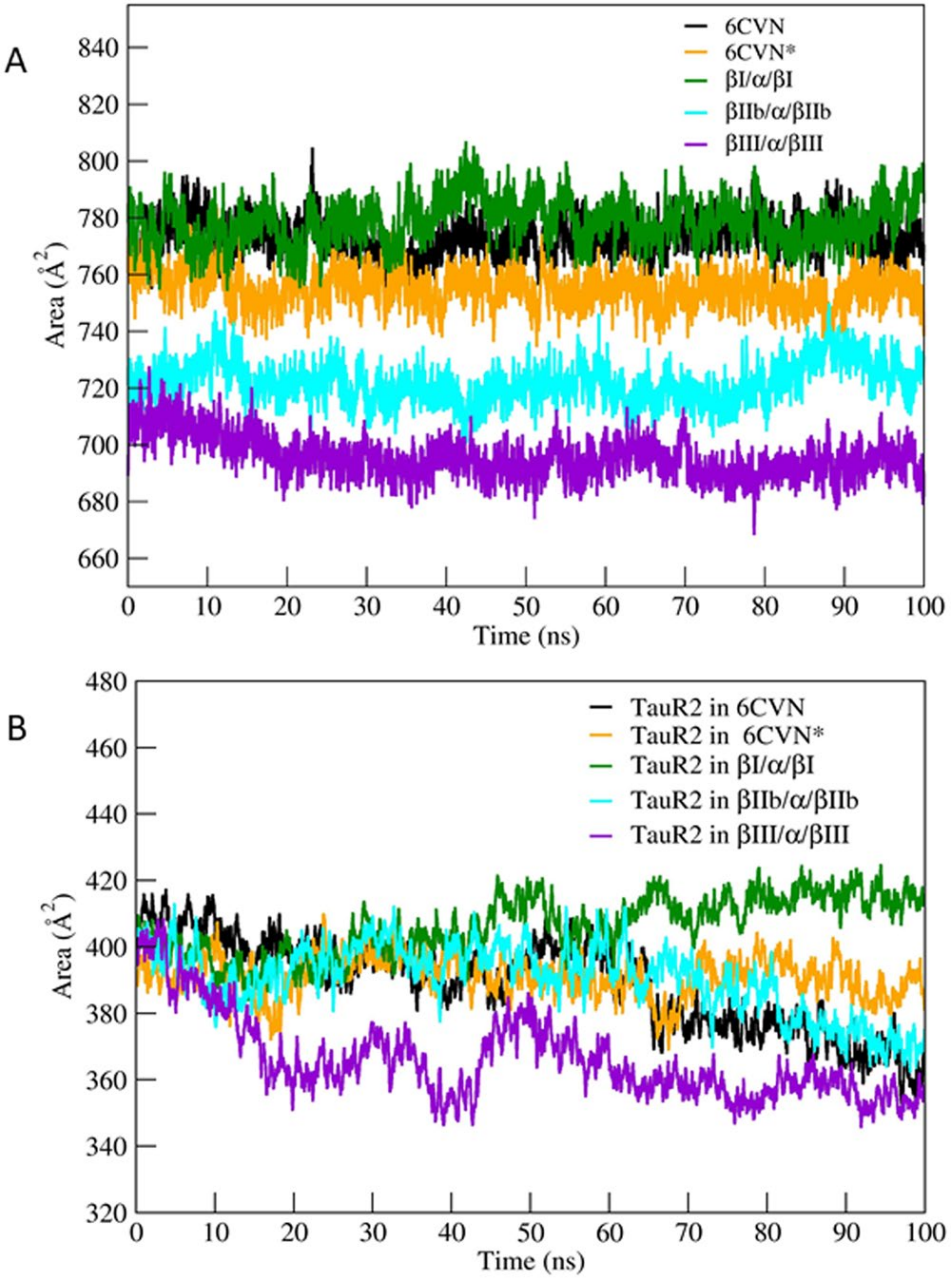
Contact surface area (CSA) and solvent accessible surface area (SASA) of different β/α/β-tubulin subunits andTauR2. (**A**) CSA for different 6CVN-TauR2 (black), 6CVN*-TauR2 (orange), βI/α/βI-TauR2 (green), βIIb/α/βIIb-TauR2 (cyan), βIII/α/βIII-TauR2 (violet) complexes. (**B**) hydrophobic SASA for tubulin isotype bound TauR2. Color scheme same as Fig. 3.

The SASA and CSA describes the accessibility of a protein surface and binding interface to the solvent respectively. It is well known that, TauR2 binds to the MT exterior surface via C-terminal tail region^7,43–46^. Therefore, we first calculated the contact surface area (CSA) of the interface where TauR2 binds, without considering flexible tail. The CSA of βIII/α/βIII is very less when compared to other tubulin isotypes (Fig. 5A) which indicates tight binding of tauR2 to the βIII/α/βIII tubulin subunits. The CSA is higher for βI/α/βI-TauR2 complex indicates weaker binding of TauR2 to the βI/α/βI tubulin subunits.

Furthermore, TauR2 bound to βIII/α/βIII tubulin subunits shows least SASA which represents tight binding of TauR2 to the βIII/α/βIII (Fig. 5B). Conversely, the TauR2 bound to βI/α/βI tubulin subunits shows higher hydrophobic SASA which indicates that exposure of hydrophobic residues is responsible for disturbed contacts between tubulin and TauR2.

Further, SASA for tubulin subunits has also been plotted and shown in Supplementary Fig. S5. The SASA for 6CVN*, βI/α/βI, βIIb/α/βIIb, βIII/α/βIII shows higher SASA values between 4900–5400 Å when compared to 6CVN-TauR2 (~4500 Å) due to the presence of C-terminal tail (Supplementary Fig. S5).

To get detailed understanding of the atomic-level interaction between tubulin isotypes and TauR2, we further analyzed hydrogen bonds formed during the course of simulation and MD simulated end-structures obtained from simulations.

### Intermolecular interactions between tubulin and TauR2 in tubulin-TauR2 complexes

In our MD simulations, the total number of hydrogen bonds formed between tubulin isotypes and TauR2 are calculated using in-built *‘gmx hbond’* tool^42^ with a cut-off value of 3.4 Å for H-bonds. All tubulin-TauR2 complexes show consistent H-bond formation throughout the simulation with the number of H-bonds roughly between 10 to 20 as shown in Supplementary Fig. S6. The details of hydrogen bonding interactions present between tubulin isotypes and TauR2 in the MD simulated end-structures are listed in Supplementary Table S4. The Supplementary Table S5 lists all the hydrophobic interactions participating in formation of stable tubulin-TauR2 complexes. Maximum number of electrostatic interactions are found in the βIII/α/βIII-TauR2 complex when compared to other tubulin-TauR2 complexes (Supplementary Table S6). Additionally, it is observed that hydrophilic interactions also have a major contribution in stabilization of tubulin subunits by TauR2 (Supplementary Fig. S7).

To further understand the role of TauR2 in stabilizing tubulin subunits, we performed secondary structure analysis of TauR2 using DSSP.

### Conformational changes in TauR2 upon tubulin binding

**Figure 6:**
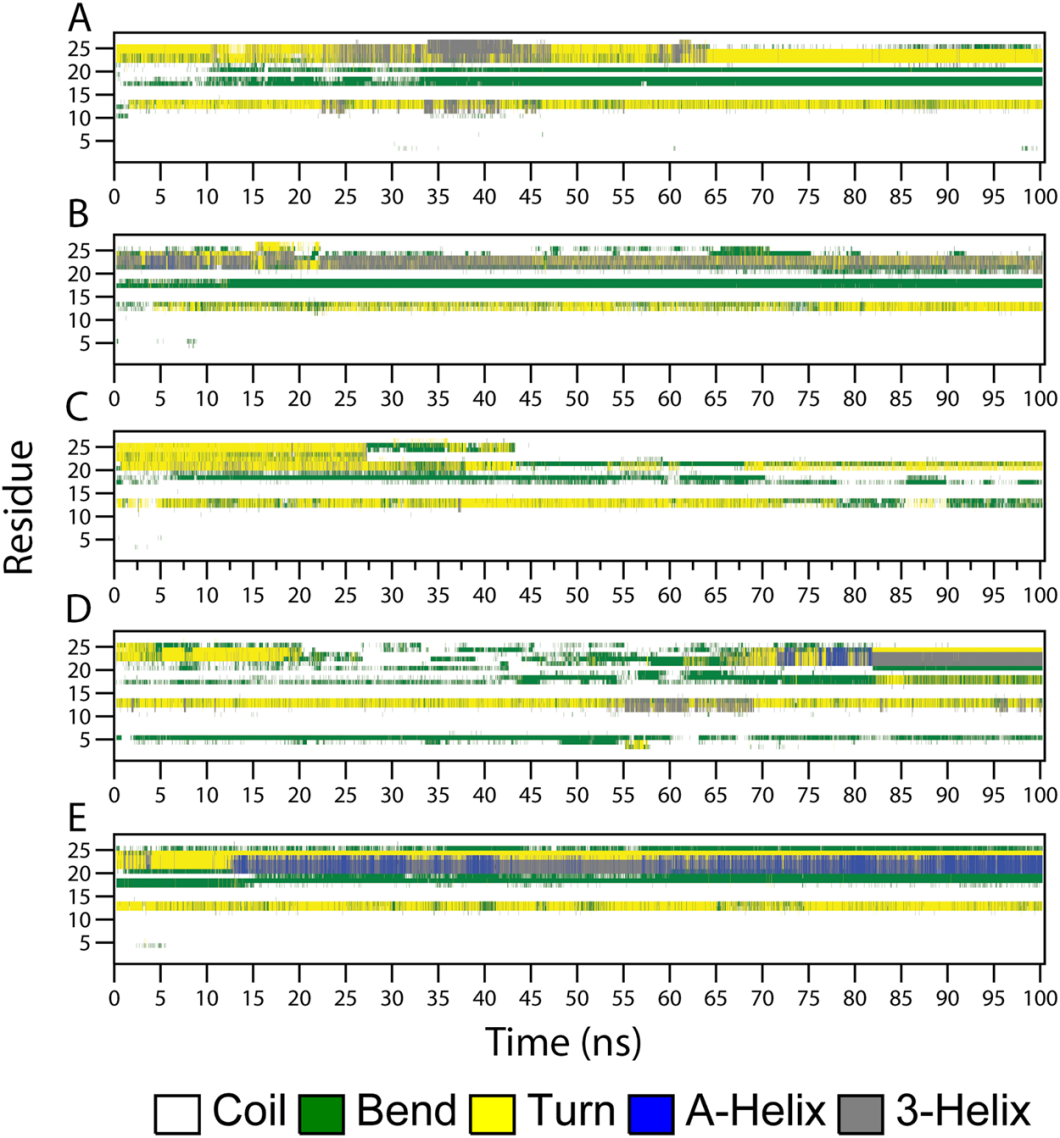
The secondary structure changes during MD simulation using DSSP for TauR2. Secondary structure changes observed in (**A**) 6CVN-TauR2 (**B**) 6CVN*-TauR2 (**C**) βI/α/βI-TauR2, (**D**) βIIb/α/βIIb-TauR2 and (**E**) βIII/α/βIII-TauR2.

Tau belongs to the class of intrinsically disordered proteins. Earlier experimental studies suggest that tau undergoes a conformational change from a disordered state to the ordered state upon binding to MT^1,47–50^. Therefore, we investigated the conformational changes in the secondary structure of TauR2 during MD simulations using DSSP program^51^. The conformational changes in the TauR2 upon binding to the tubulin subunits are shown in Fig. 6. TauR2 bound to 6CVN (Fig. 6A) and 6CVN* (Fig. 6B) show the formation of short and transient 3_10_-helix during simulation which is not very consistent throughout the course of simulation. The TauR2 bound to βI/α/βI tubulin subunits does not show the formation of either α-helix or 3_10_-helix as shown in Fig. 6C. The TauR2 in βIIb/α/βIIb-TauR2 complex exhibits the formation of short-lived α-helix and 3_10_-helix as shown in Fig. 6D. The terminal region of TauR2 (residues Ser293-Val300) undergoes a conformational transition from turn to α-helix as shown in Fig. 6E during simulation in βIII/α/βIII-TauR2 complex. We propose that, this conformational transition promotes the stable binding of TauR2 with the βIII/α/βIII tubulin subunits.

### Relative binding affinity of TauR2 towards different tubulin isotypes

The relative binding affinity of TauR2 towards tubulin subunits (β/α/β) was analyzed by calculating the binding energy of 6CVN-tau, 6CVN*-tau, βI/α/βI-tau, βIIb/α/βIIb-tau and βIII/α/βIII-tau complexes. The energy components governing the binding energy are listed in Table 1. The relative binding energy values observed for 6CVN-tau, 6CVN*-tau, βI/α/βI-tau, βIIb/α/βIIb-tau and βIII/α/βIII-tau are −927.87 ± 3.15, −1331.15 ± 3.19, −1134.13 ± 1.13, −1365.22 ± 2.26 and −1404.7 ± 1.84 kcal/mol, respectively. The binding energy analysis reveals that, interactions of βIII/α/βIII-tau complex is most favorable while 6CVN-tau complex is least favorable (Table 1). Thus, it is interesting to note the significance of C-terminal tail of the tubulin subunits in the stable binding of the tau repeat R2. The order of binding energy of TauR2 for different tubulin-TauR2 complexes is βIII/α/βIII > βIIb/α/βIIb > 6CVN* > βI/α/βI > 6CVN.

**Table 1:** The Relative binding energy of the tubulin-TauR2 complexes calculated using MMPBSA.

System	6CVN	6CVN*	βI/α/βI	βII/α/βII	βIII/α/βIII
Vdw	−97.25 ± 0.55	−131.17 ± 0.62	−133.38 ± 0.69	−124.36 ± 0.60	−125.24 ± 0.59
Elec	−1232.33 ± 3.43	−1534.36 ± 3.12	−1423.07 ± 2.77	−1659.30 ± 4.68	−1768.65 ± 2.77
Polar	413.98 ± 4.73	349.43 ± 4.18	439.27 ± 2.95	433.79 ± 4.13	505.14 ± 2.92
SASA	−12.35 ± 0.06	−15.12 ± 0.07	−17.05 ± 0.05	−15.66 ± 0.06	−16.04 ± 0.05
Binding Energy	−927.87 ± 3.15	−1331.15 ± 3.19	−1134.13 ± 1.13	−1365.2.26 ± 2.26	−1404.7 ± 1.84

All energies are given in kcal/mol.

Binding energy calculations suggest that the electrostatic interactions are favorable for binding; the βIII/α/βIII and βIIb/α/βIIb tubulin subunit show least electrostatic energy in comparison to the 6CVN and βI/α/βI tubulin subunits as shown in Table 1. Thus, the electrostatic energy makes significant contributions in the binding of TauR2 to the tubulin isotypes particularly in βIII/α/βIII-TauR2 and βIIb/α/βIIb-TauR2 complexes. The βI/α/βI-TauR2 complex exhibits higher binding energy which is responsible for its weaker affinity for TauR2.

**Figure 7:**
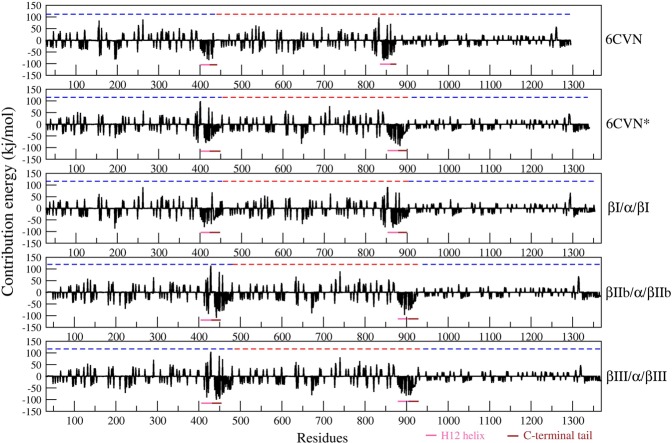
Residue decomposition energy of different tau-tubulin complexes. The H12 and C-terminal tail regions show highest energy contribution for the binding of TauR2 in 6CVN*, βI/α/βI, βIIb/α/βIIb and βIII/α/βIII tubulin subunits except in case of 6CVN which does not have C-terminal tail region.

The βIII/α/βIII-TauR2 and βIIb/α/βIIb-TauR2 complexes exhibit relatively higher affinity towards TauR2 as revealed by their relative binding energy analysis. In addition, the contribution of the individual residues in the binding energy has been analyzed by calculating the decomposition energy for each residue. The decomposition energy analysis reveals maximum contribution of H12 helix and C-terminal tail of tubulin subunits in the TauR2 binding, compared to other residues (Fig. 7). In order to highlight the importance of the interacting residues between tubulin and TauR2, per-residue interactions energy was calculated for various pairs of interacting residues between tubulin and TauR2 (Supplementary Table S7). It was found that the residues from the H12-helices and C-terminal tail regions of βIII/α/βIII complex shows maximum contribution to non-bonded contacts due to lowest energy from all pairs of interacting residues. This leads to the tight binding of the TauR2 to the βIII/α/βIII tubulin.

Hence, our relative binding energy calculations further support the results obtained from the MD simulations that the TauR2 binds stronger to the βIII/α/βIII tubulin isotype which is predominantly expressed in the neuronal cells and brain.

## Conclusion

There exists a large diversity of differently expressed β-tubulin isotypes distributed in the MT structure of different cells, which makes microtubules unique from one another in relative proportion of isotypes. The expression levels of βII and βIII has been reported to be much higher in neuronal cells (i.e., 58% and 25% respectively)^33^. In the present study, we investigated the binding mode and interaction of neuronal specific tubulin isotypes with TauR2 using molecular modeling approach.

Our MD simulation results show a stable complex formation in between different tubulin isotypes and TauR2 which are mainly mediated by the interactions of H12 helix and C-terminal tail region of the αβ tubulin isotypes with TauR2. Our results suggest that tau shows differential binding affinity towards various β-tubulin isotypes. The order of binding affinity of TauR2 with β tubulin isotypes is βIII > βIIb > βI. Thus, we find that TauR2 has greater affinity towards β tubulin isotypes which are abundantly expressed in neuronal cells i.e. βIII and βIIb. Our strategy can be potentially used to understand differential binding affinity of tau towards β tubulin isotypes present in other cell lines. We hope that knowledge of precise molecular origin of differential binding affinity of tau with β tubulin isotypes present different cell types will pave the way for developing effective treatments against tau related disorders.

## Methodology

### Sequence analysis and homology modeling of tubulin isotypes

High-resolution Cryo-EM structure of β/α/β-tubulin bound with TauR2 (PDB ID: 6CVN.pdb)^7^ was used as a template structure to build different tubulin isotype-TauR2 complexes, namely βI/α/βI-TauR2, βIIb/α/βIIb-TauR2 and βIII/α/βIII-TauR2. The template structure 6CVN.pdb is from *Sus scrofa* source with the resolution of 3.9 Å^7^. The CryoEM structure ‘6CVN.pdb’ has β/α/β-tubulin subunit bound with TauR2. The template structure (6CVN.pdb) does not have C-terminal tail, therefore we build the tail region using the Modeller 9v20 software. Hereafter, we refer to template structure with modeled C-terminal tail region as 6CVN*.

We used 6CVN.pdb as template structure for homology modeling of different human tubulin isotypes as it shows maximum identity with human βI, βIIb and βIII tubulin isotypes having uniprot IDs Q9H4B7, Q9BVA1 and Q136509 respectively. Sequence similarities between different tubulin isotypes were studied using ‘Clustal Omega’ tool^52^. The sequence analysis shows that the most residue variations are mainly at the C-terminal tail region compared to the other region.

Homology models of βI, βIIb, βIII tubulin isotypes were generated using Modeller 9v20^35^. The least energy models of β-tubulin were selected based on their discrete optimized potential energy (DOPE) score. The three dimensional stereo-chemical quality of these homology models were evaluated using Ramachandran plot through RAMPAGE^53^. The quality of the homology models were further checked using GMQE score^38^, Verify3D^39^ and ERRAT score^40^.The selected models were further used to build different tubulin isotype-TauR2 complexes such as βI/α/βI-TauR2, βIIb/α/βIIb-TauR2 and βIII/α/βIII-TauR2 using refined template structure i.e. 6CVN*-TauR2. These models (6CVN-TauR2, 6CVN*-TauR2, βI/α/βI-TauR2, βIIb/α/βIIb-TauR2 and βIII/α/βIII-TauR2) were subjected for energy minimization using Steepest Descent and Conjugate Gradient methods using Gromacs 2018.1^41^. These minimized models were used as a starting structure for molecular dynamics simulations to understand the binding mode and binding affinity of TauR2 towards different tubulin isotypes.

### Molecular dynamics simulations of tubulin-TauR2 complexes

All atom explicit molecular dynamics (MD) simulations were performed on the tubulin-TauR2 complexes (i.e. 6CVN-TauR2, 6CVN*-tau, βI/α/βI-TauR2, βIIb/α/βIIb-TauR2 and βIII/α/βIII-TauR2) using GROMACS 2018.1^41,42^. The ‘Amber99SB-ILDN’ force field^54^ was used for simulation of above-mentioned tubulin-TauR2 complexes. The force field parameter for the GDP and GTP molecules were taken from the amber parameter database^55,56^. The ‘*xleap*’ module of AmberTools was used to generate topologies and starting structure for all tubulin-TauR2 complexes^57^. These tubulin-TauR2 complexes were placed at the centre of the box with a distance of 15 Å from the wall surrounded by TIP3P water molecules with periodic boundary condition. The complexes were neutralized by adding appropriate number of required counter ions. The topology files generated using xleap module of AmberTools were converted to Gromacs topology files format using the ParmEd tool^58^. Energy minimization was performed in two steps - steepest descent algorithm was used for first 50,000 steps which was followed by 50000 steps using the conjugate gradient method^42^. The energy minimized systems were further equilibrated using canonical ensemble (NVT) followed by isothermal-isobaric ensemble (NPT). In the NVT equilibration, systems were heated to 300 K using V-rescale, a modified Berendsen thermostat^42^ for 1 ns. In NPT, all these heated systems were equilibrated using the Parrinello-Rahman barostat for 1 ns to maintain constant pressure of 1 bar. The unrestrained production MD simulations were performed for 100 ns over all the tubulin-TauR2 complexes using parameters discussed in our earlier study^59^. The long range electrostatic interactions were treated with particle mesh Ewald (PME) method^60,61^ and covalent bonds involving H-atoms were constrained using the ‘LINCS’ algorithm^62^. The time step for integration was set to 2 fs during the MD simulation. The convergence of our simulations for different tubulin-TauR2 complexes was examined using potential energy and backbone RMSD values.

We also performed all atom MD simulation on three additional systems i.e. 6CVN* (without tau), free tau and 6CVN*-polyA (as negative control) having 27 amino acids residues using same simulation protocols.

MD simulation trajectories were further analyzed by using the inbuilt tools in GROMACS 2018.1^41,42^. The secondary structure changes during MD simulation were studied using the dictionary of secondary structure of protein (DSSP) tool^51^. The simulation movies were generated using the visual molecular dynamics (VMD) software^63^ and images were generated using the Biovia Discovery studio visualizer^64^ and Chimera software^65^.

### Calculations of contact surface area for tubulin-TauR2 complexes

The SASA denotes the degree of hydration of a biomolecule which is helpful to quantify its stability in the aqueous medium. The C-terminal tail of the tubulin is highly dynamic, and it affects the overall hydrophobic SASA. Therefore, interface of tubulin trimer and TauR2 has been selected for the calculating the precise contact surface area. The in-built gromacs tool *“gmx sasa*”^66^ was used to calculate the SASA. In addition, SASA is also calculated for the tubulin subunits and the TauR2.

### Binding affinity of TauR2 towards different tubulin isotypes

The binding affinity between different tubulin isotypes and TauR2 was explored by performing relative binding energy calculation similar to earlier studies^67–69^. The stable region of the trajectory observed in between 70 ns to 100 ns and hence we extracted 70–100 ns trajectory to perform the binding energy calculations for all the tubulin-TauR2 complexes. The tool ‘g_mmpbsa’ v1.6 was used to calculate binding energy using MM/PBSA approach^70^ implemented in gromacs 2018.1. The parameters for binding energy calculations were taken from the earlier similar studies^59,69,71–73^. The binding energy (ΔG_bind_) of tubulin and TauR2 was calculated by using the following Eq. (1),

1 $${\rm{\Delta }}{G}_{bind}={\rm{\Delta }}{G}_{tubulin-TauR2}-({\rm{\Delta }}{G}_{tubulin}+{\rm{\Delta }}{G}_{TauR2})$$

Where, the ${\rm{\Delta }}{G}_{tubulin-TauR2}$, ${\rm{\Delta }}{G}_{tubulin}$ and ${\rm{\Delta }}{G}_{TauR2}$ denote the average free energies of the complex (tubulin-TauR2), receptor (tubulin) and ligand (TauR2), respectively. The calculation of entropic contribution to binding energy is computationally expensive for larger biomolecular complexes and hence it is omitted as similar to previous studies^20,21,74–76^.

## Supplementary information

Supplementary_Information

Supplementary Movie S1

Supplementary Movie S2

Supplementary Movie S3

Supplementary Movie S4

Supplementary Movie S5

Supplementary Movie S6

## Data Availability

All data generated or analysed during this study are included in this published article (and its Supplementary Information files).
