# Supplementary material for: Differential binding affinity of tau repeat region R2 with neuronal-specific β-tubulin isotypes
Source: Sci Rep. 2019 Jul 25;9:10795. doi: 10.1038/s41598-019-47249-7 (PMC6658543; doi:10.1038/s41598-019-47249-7)
Supplement: Supplementary file 1 — Supplementary_Information [file 41598_2019_47249_MOESM1_ESM.pdf]

Differential binding affinity of tau repeat region R2 with neuronal-specific β-tubulin isotypes

Vishwambhar Vishnu Bhandare, Bajarang Vasant Kumbhar and Ambarish Kunwar*

Department of Biosciences and Bioengineering, Indian Institute of Technology Bombay, Powai, Mumbai-400076, Maharashtra, India

*Corresponding author

E-mail: akunwar@iitb.ac.in

Supplementary Figures

Supplementary Figure S1. Variation of potential energy with time for 6CVN-TauR2 (black), 6CVN*-TauR2 (orange), βI/α/βI-TauR2 (green), βIIb/α/βIIb-TauR2 (cyan), βIII/α/βIII-TauR2 (violet) showing that potential energy of the simulated Tubulin-TauR2 complexes were stable during the course of simulation.

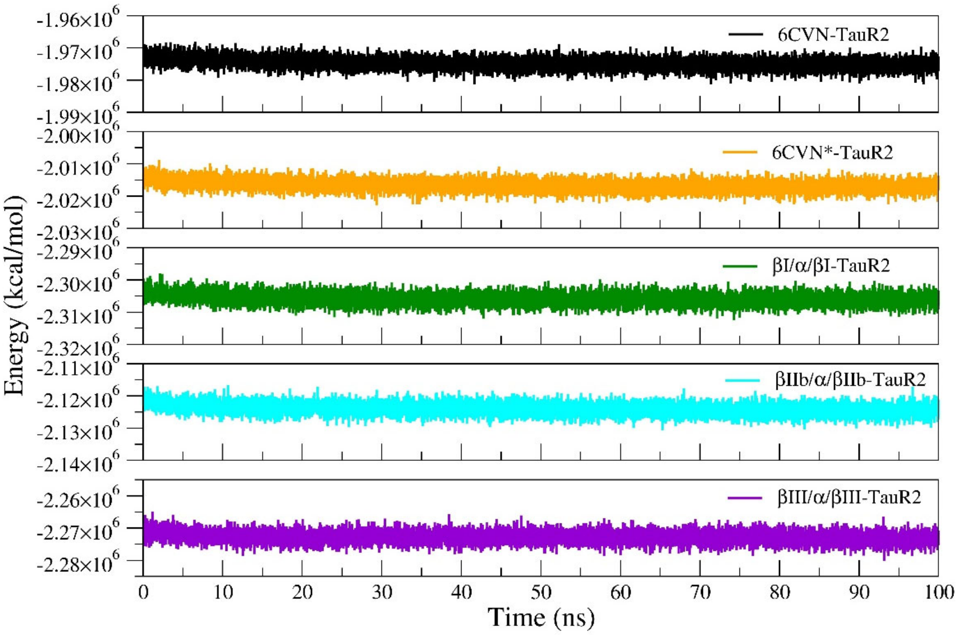

Supplementary Figure S2. Backbone Root mean square deviation for different tubulin subunits. RMSD values are shown for 6CVN (black), 6CVN* (orange), βI/α/βI (green), βIIb/α/βIIb (cyan) and βIII/α/βIII (violet).

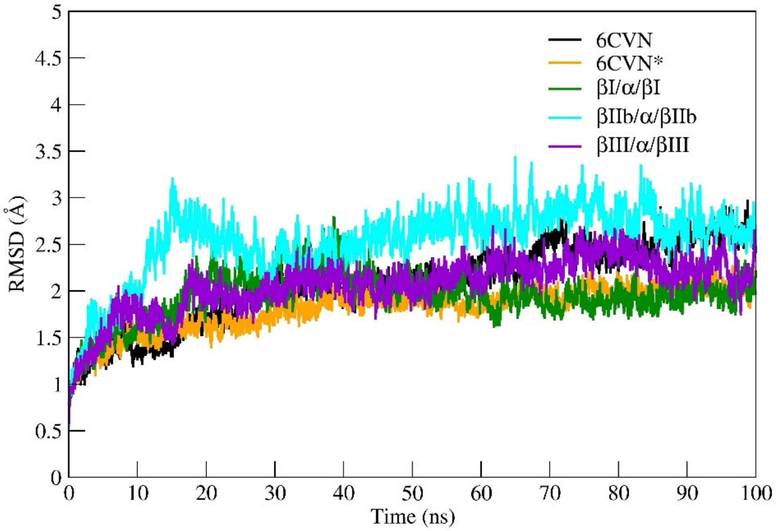

Supplementary Figure S3. Root mean square fluctuations (RMSF) of different β/α/β tubulin subunits and TauR2 **(A)** RMSF of different β/α/β tubulin subunits **(B)** Magnified view of their C-terminal H12 helix and tail regions **(C)** RMSF of TauR2 bound with different β/α/β tubulin subunits observed during the simulations5. Color scheme is same as Supplementary Figure S2**.**

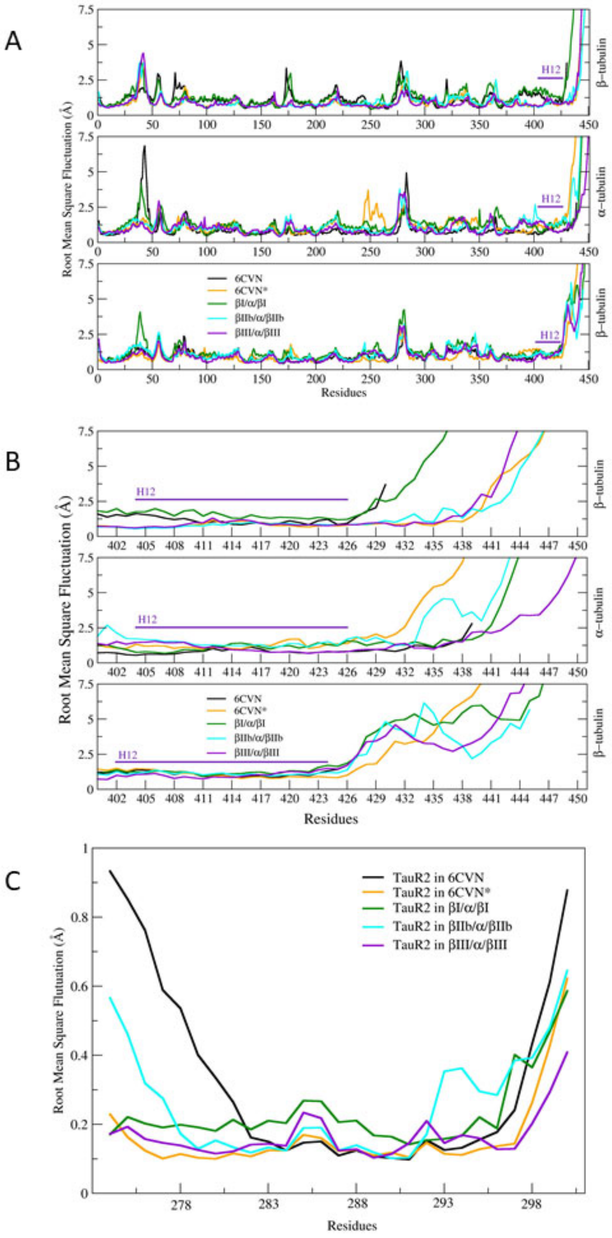

Supplementary Figure S4. Radius of Gyration for different tubulin isotypes. Color scheme is same as Supplementary Figure S2**.**

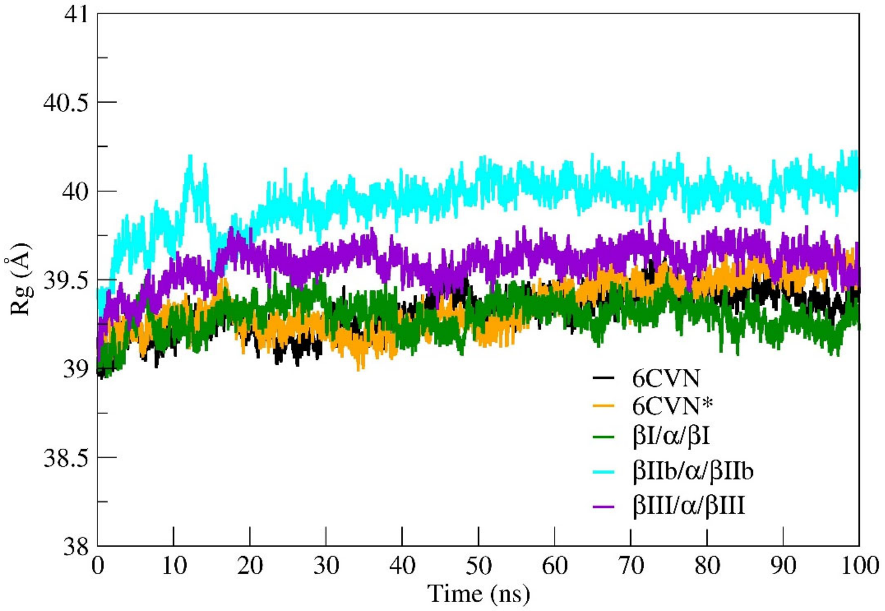

Supplementary Figure S5. Solvent accessible surface area for different tubulin subunits. SASA plotted for 6CVN (black), 6CVN* (orange), βI/α/βI (green), βIIb/α/βIIb (cyan), βIII/α/βIII (violet) are shown. Color scheme is same as Supplementary Figure S2**.**

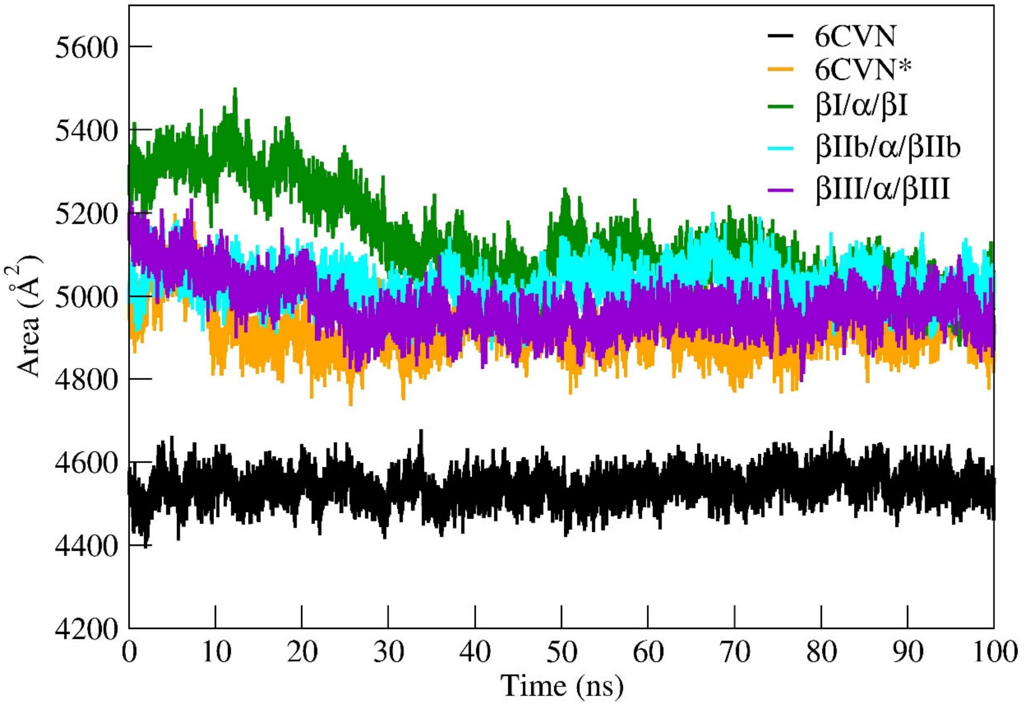

Supplementary Figure S6. The number of hydrogen bonds formed in between tubulin subunits and TauR2 during MD simulation. Color scheme is same as Supplementary Figure S1**.**

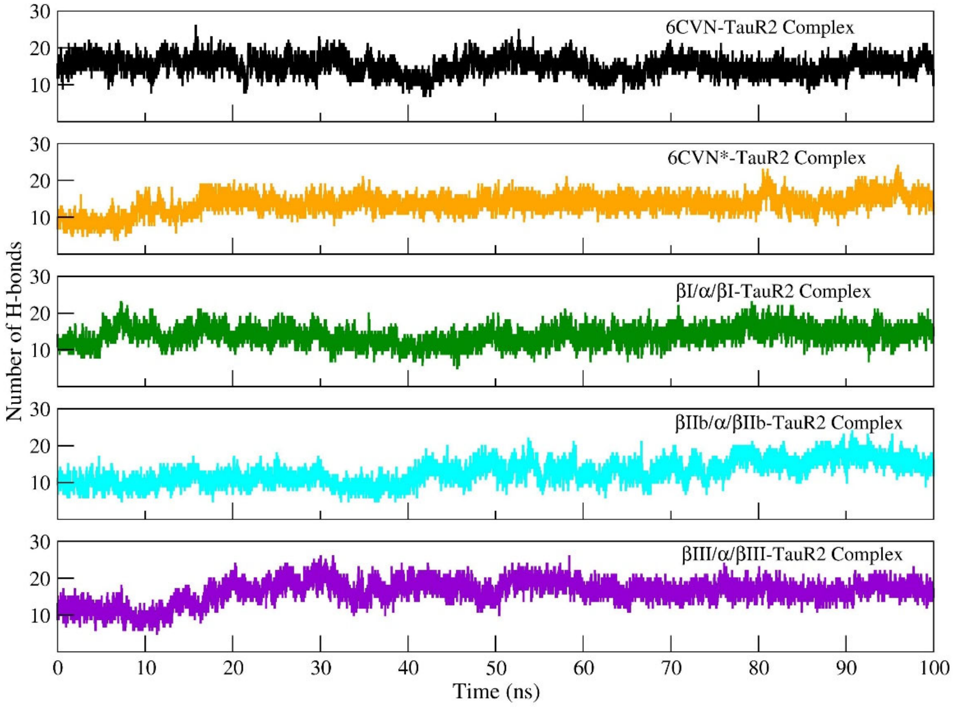

Supplementary Figure S7. Analysis of MD simulated end-structures. Molecular dynamics simulated end-structures of tubulin-TauR2 complex. The scale represents the amplitude of color gradient ranges from blue (hydrophilic) to red (hydrophobic). Stable complex formation in (A) 6CVN and TauR2, (B) 6CVN* and TauR2, (C) βI/α/βI tubulin subunit and TauR2, (D) βIIb/α/βIIb tubulin subunit and TauR2, (E) βIII/α/βIII tubulin subunit and TauR2 are mediated by hydrophilic interactions.

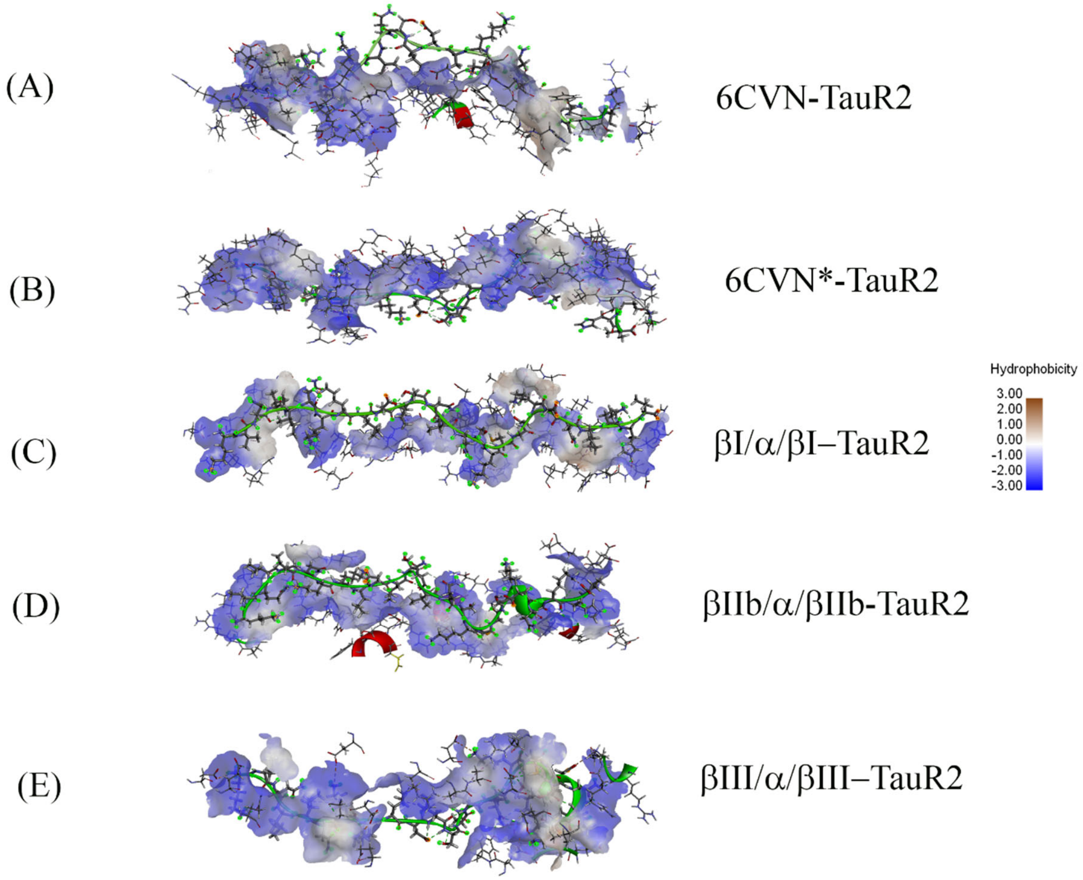

Supplementary Tables

Supplementary Table S1. DOPE score for the selected homology models of Chain A and C of βI, βIIb and βIII tubulin isotypes used in simulations.

Isotype	DOPE score	
	chain A	chain C
βI	-54.299.89	-54291.24
βIIb	-53487.13	-53054.42
βIII	-53725.86	-53054.42

Supplementary Table S2. Percentage and number of residues present in the favored, allowed and outlier regions of Ramachandran Plot for homology models of tubulin isotypes.

Region	β1 tubulin		β2 tubulin		β3 tubulin	
	Percentage (No. of residues)	Percentage (No. of residues)	Percentage (No. of residues)	Percentage (No. of residues)	Percentage (No. of residues)	Percentage (No. of residues)
	**chain A**	**chain C**	**chain A**	**chain C**	**chain A**	**chain C**
Favored regions	98.4 (442)	98.9 (444)	98.9 (443)	98.9 (438)	98.9 (443)	98.9 (443)
Allowed region	1.3 (6)	0.9 (4)	0.7 (3)	1.1 (5)	1.1 (5)	0.7 (3)
Outlier region	0.2 (1)	0.2 (1)	0.4 (2)	0 (0)	0 (0)	0.4 (2)

Supplementary Table S3. Validation of three-dimensional models of chain A and chain C generated for βI, βIIb and βIII isotypes using Swiss model GMQE score, Verify-3D and Errat score.

Score	βI		βIIb		βIII	
	chain A	chain C	chain A	chain C	chain A	chain C
GMQE	0.98	0.98	0.98	0.98	0.98	0.98
Verify3D	93.13%	92.02%	98.43%	98.43%	98.44%	92.67%
Errat	81.32	83.26	87.47	83.30	86.36	83.33

Supplementary Table S4. Hydrogen bonding interaction between tubulin subunits and TauR2 after molecular dynamics simulations.

System	Atoms involved in H-bonding	Distance (Å)	Angle (°)
6CVN-TauR2	D:SER16:HG - B:GLU434:OE2	1.55968	170.912
	C:LYS392:HZ2 - D:ASP22:OD1	1.79981	155.003
	D:SER20:H - B:GLU434:OE1	1.80025	149.621
	D:CYS18:H - B:ASP431:OD1	1.8071	147.422
	D:GLY19:H - B:ASP431:OD1	1.81516	170.051
	D:ASN23:HD21 - C:PHE389:O	1.83614	168.422
	D:ILE5:H - B:GLU415:OE1	1.87843	148.138
	B:ARG402:HH22 - D:LYS7:O	1.95256	131.998
	D:LYS21:HZ3 - B:ASP438:O	1.96835	128.744
	C:ARG391:HE - D:SER20:O	1.97398	162.451
	D:ASN6:H - B:GLU415:OE2	1.97803	165.667
	D:LYS17:HZ2 - B:ASP424:OD1	2.01656	167.691
6CVN*-TauR2	A:SER16:HG - E:ASP431:OD2	1.56368	161.89
	A:ASN6:HD21 - G:GLN433:OE1	1.68122	165.335
	A:LYS1:HZ1 - G:ASP417:OD1	1.73491	152.719
	A:LYS7:HN - E:ALA400:O	1.75148	168.412
	A:SER12:HG - A:ASP10:OD2	1.77457	163.204
	E:LYS401:HZ1 - A:ASN6:OD1	1.77792	156.289
	A:SER20:HN - E:GLU434:O	1.8254	168.05
	A:ILE4:HN - G:GLN424:OE1	1.85744	159.004
	F:ARG391:HH21 - A:SER20:OG	1.88141	150.648
	A:LYS17:HN - E:ASP431:OD2	1.88237	156.579
	A:LYS1:HT2 - G:ASP417:OD2	1.93321	163.221
	A:LYS25:HZ2 - A:VAL27:OXT	1.95566	155.991
	F:ARG391:HE - A:SER20:O	1.95954	137.171
	A:SER12:HN - A:ASP10:OD2	1.97395	166.966
	A:VAL2:HN - G:GLU421:OE2	1.97952	167.491
	A:SER20:HG - E:TYR262:OH	2.01579	158.876
	A:CYS18:HN - E:ASP431:OD2	2.04985	143.832
	A:ASP22:HN - A:ASP22:OD1	2.06458	123.428
βI/α/βI-TauR2	D:SER20:HG - B:GLU434:OE2	1.71015	154.981
	D:LYS17:H - B:ASP431:OD2	1.73879	154.124
	D:SER16:HG - B:ASP431:OD2	1.75545	159.778
	D:VAL2:H - A:GLU421:OE1	1.79605	175.632
	D:SER20:H - B:GLU434:OE2	1.79906	165.806
	D:LYS21:HZ1 - B:GLU434:O	1.82861	143.493
	B:LYS430:HZ2 - D:VAL14:O	1.84508	147.656
	D:ASN23:HD22 - C:PHE389:O	1.87483	147.025
	D:LYS1:H3 - A:ASP417:OD1	1.96013	159.665
	D:LYS7:H - B:ALA400:O	2.03926	154.297

	D:ILE4:H - A:GLN424:OE1	2.05966	141.181
**βIIb/α/βIIb-TauR2**	A:SER16:HG - E:ASP431:OD1	1.71023	174.429
	A:LYS17:HZ2 - E:ASP424:OD2	1.72125	162.879
	A:LYS1:HT2 - F:GLU421:OE2	1.73767	160.093
	A:LYS25:HZ2 - E:GLU445:OE1	1.7524	156.386
	A:LYS7:HN - E:ALA400:O	1.7726	158.577
	A:ASN6:HD22 - F:ASP431:OD1	1.88399	166.501
	A:LYS17:HN - E:ASP431:OD1	1.90489	145.148
	E:ARG402:HH12 - A:LYS7:O	1.90591	147.259
	A:VAL27:HN - E:GLU445:OE1	1.92907	160.86
	A:CYS18:HN - E:ASP431:OD1	2.03765	145.506
	A:LYS25:HZ3 - E:GLU446:O	2.04646	165.654
	F:GLN424:HE21 - A:LYS1:O	2.06218	173.384
**βIII/α/βIII-TauR2**	A:SER16:HG - E:ASP431:OD1	1.57372	172.137
	A:LYS21:HZ1 - E:GLU443:OE2	1.74362	173.969
	F:GLN424:HE21 - A:VAL2:O	1.79549	177.625
	A:GLN15:HE21 - E:GLU443:OE2	1.81621	154.684
	A:LYS8:HZ3 - F:GLU433:OE1	1.84962	164.797
	A:LYS8:HZ1 - E:ASP396:OD1	1.85984	168.252
	G:ARG391:HH11 - A:ASN23:OD1	1.89423	162.494
	E:GLY442:HN - A:ILE24:O	1.93324	171.538
	A:LYS17:HN - E:ASP431:OD1	1.99492	142.974
	A:CYS18:HG - E:ASP431:OD1	2.0734	155.708

Supplementary Table S5. Hydrophobic Interactions between different β/α/β-tubulin isotypes and TauR2 after molecular dynamics simulations.

System	Hydrophobic Interactions	Distance (Å)
**6CVN-TauR2**	B:ALA427 - D:LYS17	4.23972
	B:ARG264 - D:CYS18	4.32305
	B:ALA426 - D:VAL14	4.34456
	B:ARG402 - D:ILE4	5.12692
	B:VAL409 - D:ILE4	5.16258
	B:ARG422 - D:LEU11	5.2126
	B:ALA427 - D:VAL14	5.2724
**6CVN*-TauR2**	E:ALA426 - A:VAL14	3.78619
	E:ALA426 - A:LEU11	4.1983
	E:ALA400 - A:LYS8	4.29681
	G:PHE260 - A:ILE4	4.3943
	A:VAL2 - G:PRO261	4.85577
	A:LEU9 - A:LEU11	5.10635
	A:LYS21 - E:VAL437	5.11425
	E:ALA427 - A:VAL14	5.28218
	A:VAL14 - A:LEU11	5.3507
	E:ARG422 - A:LEU9	5.37331
	A:VAL2 - A:ILE4	5.42169
**βI/α/βI-TauR2**	B:ALA426 - D:LEU11	4.086
	B:ALA426 - D:VAL14	4.25902
	A:PHE425 - D:ILE4	4.29071
	B:ALA427 - D:VAL14	4.57533
	A:PHE260 - D:VAL2	4.62894
	B:TYR262 - D:CYS18	4.75533
	B:PRO263 - D:LYS17	4.89022
	B:ARG402 - D:LYS7	4.99921
	D:CYS18 - B:VAL435	5.09522
	B:ARG422 - D:LEU9	5.15149
	B:LYS430 - D:VAL14	5.38523
	A:ALA428 - D:ILE4	5.40848
	B:VAL440 - D:LYS21	5.41789
	C:ILE405 - D:ILE24	5.46457
**βIIb/α/βIIb-TauR2**	E:ALA427 - A:LYS17	3.91294
	E:ALA426 - A:VAL14	3.93471
	E:ALA426 - A:LEU11	4.19609
	A:CYS18 - E:ARG264	4.36259
	E:ALA400 - A:LYS8	4.4046
	A:CYS18 - E:PRO263	5.04073
	A:CYS18 - E:ILE265	5.21553
	G:LYS392 - A:ILE24	5.27213
	E:TYR399 - A:LEU9	5.32769
**βIII/α/βIII-TauR2**	E:ALA426 - A:LEU11	3.83867
	E:ALA426 - A:VAL14	4.14469
	E:ALA427 - A:LYS17	4.22472
	E:ALA427 - A:VAL14	4.83218
	E:ARG422 - A:LEU11	4.84097
	A:LYS25 - E:VAL437	5.06163
	A:CYS18 - E:ARG264	5.14951
	E:ARG422 - A:LEU9	5.48905

Supplementary Table S6. Electrostatic Interactions between different β/α/β-tubulin isotypes and TauR2 after molecular dynamics simulations.

Systems	Electrostatic interactions	Distance (Å)
**6CVN-TauR2**	D:LYS8:NZ - A:ALA430:O	4.04432
	D:LYS21:NZ - B:SER439:O	4.66847
	D:LYS25:NZ - B:GLU434:OE2	4.90999
	D:LYS21:NZ - B:GLU434:OE1	5.38615
**6CVN*-TauR2**	A:LYS1:N - G:GLU421:OE2	4.85022
	A:LYS7:NZ - E:GLU415:OE1	4.9846
	A:LYS25:NZ - E:GLU441:OE1	5.12197
	A:LYS17:NZ - E:ASP424:OD2	5.27557
**βI/α/βI-TauR2**	D:LYS1:N - A:GLU421:OE1	2.86182
	D:LYS25:NZ - C:GLU412:OE1	4.31321
	D:LYS7:NZ - B:GLU415:OE1	4.45715
	D:LYS21:NZ - B:GLU434:OE2	4.75044
**βIIb/α/βIIb-TauR2**	A:LYS21:NZ - E:GLU434:OE2	4.3529
	A:LYS8:NZ - E:ASP396:OD2	5.021
**βIII/α/βIII-TauR2**	A:LYS25:NZ - E:GLU434:OE2	2.68494
	A:LYS25:NZ - E:GLU450:OE2	2.85019
	A:LYS1:N - F:ASP417:OD2	2.91844
	A:LYS1:N - F:GLU421:OE2	4.28381
	A:LYS7:NZ - E:GLU415:OE1	4.31182
	A:LYS21:NZ - E:GLU434:OE1	4.48844
	A:LYS17:NZ - E:ASP424:OD2	4.70401
	G:LYS392:NZ - A:ASP22:OD2	4.95323
	A:LYS21:NZ - E:GLU450:OE2	5.37409

Supplementary Table S7. Per residue interaction energy (in kJ/mol) calculated for residues involved in H-bonding, hydrophobic and electrostatic interactions for different β/α/β tubulin subunits of different Tubulin-TauR2 complexes. Residues belonging to β tubulins of chain A and chain C are shown in first and third row respectively with blue background. Residues belonging to α tubulins of chain B are shown in second row with red background. The residues with negative energy values (white) are favourable for binding of TauR2 while residues with positive energy values (sky blue) are unfavourable.

**6CVN-TauR2**		**6CVN*-TauR2**		**βI/α/ βI-TauR2**		**βIIb/α/βIIb-TauR2**		**βIII/α/βIII-TauR2**	
Residues	Energy	Residues	Energy	Residues	Energy	Residues	Energy	Residues	Energy
Ala430	-73.27	Glu421	-18.28	Glu421	-56.18	Glu421	-73.59	Asp417	-72.28
		Glu415	-81.28	Asp417	-11.28	Asp431	-73.27	Glu421	-80.71
		Glu441	-29.21	Gln424	0.10	Gln424	-1.96	Glu433	-71.18
		Asp424	-64.48	Phe260	-0.18			Gln424	2.22
		Ala426	-4.35	Phe425	0.03				
		Ala400	-5.11	Ala428	0.10				
		Phe260	-0.02						
		Pro261	-0.10						
		Asp417	-19.91						
Glu434	-32.99	Val437	-5.86	Glu434	-17.29	Asp431	-52.33	Asp431	-65.25
Asp431	-50.99	Ala427	-5.34	Asp431	-12.28	Asp424	-61.19	Glu443	-50.41
Glu415	-70.51	Arg422	69.72	Lys430	71.27	Glu445	-13.85	Asp396	-75.05
Asp438	-48.20	Tyr262	-7.30	Ala400	-4.42	Ala400	-10.47	Gly442	-4.42
Asp424	-56.68	Asp431	-46.55	Ala426	-3.68	Arg402	108.98	Ala426	-2.31
Ala427	-7.95			Ala427	-2.71	Glu446	-40.08	Ala427	-5.39
Ala426	-5.08			Tyr262	-6.64	Ala427	-7.95	Arg422	83.45
Val409	-4.74			Pro263	-4.39	Ala426	-3.76	Arg264	69.60
Arg422	64.74			Arg402	89.47	Arg264	60.27	Val437	1.51
Arg402	96.91			Val435	-4.07	Ile265	-0.44	Glu434	-46.44
Ser439	-49.43			Arg422	65.06	Tyr399	-5.15	Glu450	-44.63
Arg264	55.84			Val440	-3.18	Asp396	-88.00	Asp424	-75.12
				Glu415	-83.54	Glu434	-61.42		
Lys392	42.98	Arg391	37.41	Phe389	0.71	Lys392	58.28	Arg391	38.19
Phe389	-0.12			Ile405	-5.06			Lys392	40.22
Arg391	54.52			Glu412	0.35				
**Total =**	**-84.97**	**Total =**	**-180.66**	**Total =**	**12.19**	**Total =**	**-265.93**	**Total =**	**-358**

Supplementary Movies

Supplementary Movie S1. The interactions of TauR2 with 6CVN during MD simulation.

Supplementary Movie S2. The interactions of TauR2 with 6CVN* during MD simulation.

Supplementary Movie S3. The interactions of TauR2 with βI/α/βI during MD simulation.

Supplementary Movie S4. The interactions of TauR2 with βIIb/α/βIIb during MD simulation.

Supplementary Movie S5. The interactions of TauR2 with βIII/α/βIII during MD simulation.

Supplementary Movie S6. The interactions of polyA (negative control) with 6CVN* during MD simulation.
